# Benefits of Veterinary Herd Health Management on German Dairy Farms: Status Quo and Farmers' Perspective

**DOI:** 10.3389/fvets.2021.773779

**Published:** 2022-01-11

**Authors:** Jenny Ries, Katharina Charlotte Jensen, Kerstin-Elisabeth Müller, Christa Thöne-Reineke, Roswitha Merle

**Affiliations:** ^1^Department of Veterinary Medicine, Institute for Veterinary Epidemiology and Biostatistics, Freie Universität Berlin, Berlin, Germany; ^2^Ruminant and Swine Clinic, Department of Veterinary Medicine, Freie Universität Berlin, Berlin, Germany; ^3^Department of Veterinary Medicine, Institute of Animal Welfare, Animal Behavior and Laboratory Animal Science, Freie Universität Berlin, Berlin, Germany

**Keywords:** survey, integrated herd health management, latent class analysis, satisfaction with veterinarian, decision-making

## Abstract

Veterinary Herd Health Management plays an important role in veterinary medicine on dairy farms and has also been mandatory at the European Union level since April 21, 2021. Despite the increasing importance of VHHM, little is known about the extent of utilization of VHHM by dairy farmers and their view on this type of collaboration. Therefore, this cross-sectional study aimed to determine the status quo of the currently practiced VHHM in Germany. For this purpose, an online survey was conducted among dairy farmers in November and December 2020. From 216 analyzed questionnaires, about half (*n* = 106) of the surveyed dairy farmers used VHHM at different scopes. However, regardless of the group, the term “veterinary herd health management” generally was given most relative importance by the participants as a veterinary service for herd fertility improvement, rather than the actual definition of a holistic approach. In contrast to this, the actual motivation of the VHHM participants, to take part in such a program was primarily based on the desire to safeguard animal health by employing preventive measures, that is, to avoid the occurrence of diseases *via* improved management and to improve farm performance (and profitability). Dairy farmers who opted for VHHM tended to manage larger higher yielding herds than those who did not. Additionally, the farmers in latter farms were more likely to make joint animal health decisions with their veterinarians. Using a latent class analysis, two groups of farmers among farms that were not currently using VHHM were identified, one of which expressed great interest in using VHHM while the other did not. Since the new legal basis makes the topic even more relevant than before, dairy farmers, animals, and veterinarians might benefit from the study to exploit hidden opportunities for VHHM collaboration.

## Introduction

German dairy farming is undergoing tremendous structural changes. The constant intensification in this field over the last few years is reflected in the decrease in the number of dairy farms between 2010 and 2020 by almost 40% (2020: 54,300 farms) and the decrease in the number of animals by almost 6% (2020: ~3.9 million dairy cows). As a result, the average number of dairy cows per farm increased from 46 to 72 (1). At the same time, there is an increasing call for sustainability in favor of animal welfare and the protection of natural and social resources (2). Ensuring sufficient food production under changing conditions and an intensified societal focus also plays a central role in the latest national development, with the collaboration of the German government's Commission on the Future of Agriculture (3). Therefore, it is even more understandable that, not only partly due to the ever-increasing societal pressure (2, 4, 5), but also due to legal and economic constraints, the focus of veterinary activity is shifting significantly from therapy to prevention. In 1994, researchers described the four phases of preventive veterinary medicine in animal husbandry, with phase three, which involves the application of proactive rather than reactive measures, established in the 1960s (6). Back then, prophylaxis focused particularly on fertility or udder health, and subclinical diseases were recognized for the first time as an obstacle to increased productivity. In this context, farmers began to pay for veterinary consulting services. Since then, veterinarians have become increasingly important as advisors on dairy farms (7, 8).

The highly topical nature of preventive herd management lies in the fact that VHHM has recently become legally binding. Until April 21, the 2016/429 Regulation (the so-called “EU Animal Health Law”) had to be implemented in the European Union (EU) member states (9). Therefore, it is no longer a question of whether such models have a part in the future in Germany, but rather what their structure will look like (10, 11). More than 10 years ago, <6% of farmers participated in VHHM (12); in the future, all of these farmers are expected to participate in VHHM programs. In addition, VHHM is an integral part of animal food production in other countries such as the Netherlands (13, 14), Austria (15), Denmark (16), Sweden (17), the United Kingdom (18), and Canada (19).

Whether VHHM can be fully implemented is additionally influenced by an important factor: the increasingly severe shortage of livestock-focused veterinarians. The fact that the next generation of veterinarians is even more transient to this field of practice than it already is compared to veterinarians working on other animal species, does not favor the provision of high-quality veterinary herd care on farms (20, 21).

Regardless of the extrinsic factors mentioned previously, a farmer's intrinsic motivation plays a major role in the decision to implement or not implement VHHM (22, 23). Compared to some audit systems in Germany, such as quality management milk (QM) standard or dairy internal programs (24, 25), that allow farms to develop a direct financial dependency with immediate negative consequences in case of non-compliance (e.g., milk revenue is influenced by farm individual sustainability and animal welfare measures), VHHM is not meant for achieving that kind of hierarchical control. Rather, it is intended to encourage equal cooperation between the farmers and the veterinarian and to guide the farmers, free of premiums, using a mature concept with the help of a veterinarian, with a focus toward improved animal health and animal welfare, which in turn positively improves profitability. Several studies have already proven that the attitude and character of farm managers have a major influence on the implementation and progress of a herd management program (15, 16, 23). In addition, success depends on a good veterinarian-livestock owner relationship and interpersonal communication (26–29). Although the veterinarian is considered one of the most important external advisors on the farm (30) and livestock owners value him or her for, for example, up-to-date expertise or information on industry-relevant events (26, 31, 32), this alone does not guarantee a successful collaboration. Researchers found that, according to farmers, the main criteria for implementing the veterinarian's suggestions are trust, practicability, and agreement with their own priorities (23, 33). The veterinarian must be aware of these criteria; otherwise, only 50% of the veterinarian-suggested changes will be applied (33). If the basic conditions for successful cooperation are in place, other positive effects have been proven on VHHM farms: studies showed a correlation between VHHM participation and loose housing as well as a higher degree of digitalization, which is potentially associated with higher levels of herd performance (26, 30, 34, 35) and possibly better animal welfare (4, 12).

Of course, VHHM not only has advantages; the additional costs (13, 26, 34) and time associated with a visit of the veterinarian, including a tour around the farm, should not be ignored (12, 36).

This study was divided into two separate parts: The here present aimed to give an insight from the farmers' perspective on the implementation and practices of VHHM on German dairy farms. The focus was to determine the extent to which the VHHM is implemented on German farms and how this is shaped. Dairy farmers in Germany were asked about their attitude toward and satisfaction with VHHM to enable veterinarians to develop better farm-specific, and thus successful, concepts. While this is more concerned with the “soft skills” of a potential VHHM program, the second publication deals with the “hard facts,” meaning the association between farm performance and (non-) participation in a VHHM program. It was expected that in the group of VHHM farms, a higher satisfaction with the veterinarian and the VHHM program would be accompanied by a better overall farm performance.

## Materials and Methods

### Study Design

The online survey tool Lime Survey® (LimeSurvey GmbH, Hamburg, Germany) was used to collect data for this cross-sectional study. The study was conducted from November 1 to December 31, 2020.

### Questionnaire Design

A questionnaire comprising 123 questions was created based on a study conducted in the Netherlands (37). The underlying questionnaire contained a total of 10 subgroups of questions: All participants were asked 40 questions from different groups, as described below. Question group 3 divided participants into VHHM-participants and non-VHHM-participants. The non-VHHM participants were asked another 9 questions, while the VHHM-participants had another 74 questions. Since the answers given previously were used to decide which additional questions should be administered, neither of the participants had to answer all 123 questions. Estimated by pre-testing, it took the non-VHHM participants ~12 min to complete the questionnaire and the VHHM participants 20 min.

The questionnaire consisted largely of closed questions with a single choice and contained questions that are rated on a 5-point Likert scale. Open-ended questions were used less frequently as well as ranking questions. The first page contained a detailed explanation of the goals and processes of the survey. At the end of the introduction, the participants were provided with a privacy notice from the Institute of Veterinary Epidemiology and Biostatistics, Freie Universität Berlin, and had to agree to a data processing consent form.

In the first block, general farm data were collected. The number of animals (whole herd including young stock vs. lactating and dry cows of the herd), 305-day milk yield, daily milk yield, milk fat and protein content, bulk tank somatic cell count (BTSCC) of the last two inspections during the DHI (Dairy Herd Improvement) testing (also known as MLP in Germany) or analysis of the dairy product, age at first calving (AFC), and replacement rate (RR) were included in the statistical analyses. Farm type, animal breed, management type, housing type, bed type, use of automatic milking systems (AMS), participation in “MLP” testing, and number of cows that died in <60 days in milk (DIM) were also asked.

The second section assessed in more detail the resources of the available labor force. Of importance to us were the number and expertise of the workforce, employment model, in-house communication, and employment of foreign language workers.

As regards VHHM, all participants were asked questions about their relative importance of subjective VHHM definition, their participation in VHHM, their participation in animal health decision-making, and overall satisfaction with their veterinarian. The answers given were used to decide which additional questions should be administered. Those who did not use VHHM at the time of the survey were asked about possible past participation in a VHHM program and whether they saw a potential need for it in their farm. They were also asked about their willingness to pay for veterinary consultation and their opinion on the offer provided by their cooperating veterinarians. The final set of questions for this group was related to the use of non-veterinary consultants on the farm.

Participants who indicated to receive VHHM support for their farm were asked questions about the detailed design of the service. First, two questions about motivation of participation in VHHM were asked, of which the first one was a free-text field. After this, the second question provided answers that had to be rated on a 5-point Likert Scale (1 = fully applicable; 5 = not applicable at all). Further collaboration was analyzed by asking the participants about perceived advantages and disadvantages of VHHM, and cooperation with the involved veterinarian, which was also assessed with a 5-point Likert Scale. Subsequently, a filter question about the VHHM components, as in which field support was received, was asked. Depending on these answers, the question about “fulfillment of expectations” was asked, with answers rated by school grades (German school grades: 1 = very good to 5 = insufficient). Additionally, the section “Future of the VHHM” with questions about the opportunities for improvement in the components covered in VHHM as well as the precise content of each VHHM component was assessed in detail. Moreover, the accounting for VHHM was examined by asking the participants to answer questions related to the current billing and desired billing method.

Questions regarding the demographics of the participants were indicated at the end of the questionnaire. According to their geographical location, the federal states were grouped into four major regions: North, East, South, and West of Germany. The northwestern part is characterized by family-run business. Dairy farms in the East are characterized by their corporate structure with numerous employees and bigger in size, due to having been the former German Democratic Republic. In southern Germany, the average herd size is smallest, and these family-run farms are still very traditional.

A two-phase pretest of the questionnaire was conducted prior; in phase I, three dairy farmers were asked to complete the questionnaire survey in the presence of the first author to determine whether the farmers understood the content of the questionnaire and the answer options provided. If necessary, questions were adapted, and more detailed explanations were added. In phase II, three additional dairy farmers were selected to complete the questionnaire in an online format without prior explanation, while the first author recorded and documented the time required. Comprehension problems were no longer observed in this phase, but a few questions were shortened so that the limit of the survey could be realized.

### Participants

Participation in the survey was voluntary and only possible online *via* an online link. The survey was not limited to a certain region, and all farmers' associations (“Deutscher Bauernverband;” 1 head association with 18 regional associations) were asked to disseminate the link among their members. Furthermore, additional associations such as the “Bundesverband der Maschinenringe e.V.” (with all 248 sub-associations), “Bund Deutscher Milchviehhalter e.V.,” “Deutscher Raiffeisenverband e.V.,” and “Bund der deutschen Landjugend e.V.” were contacted by mail and asked for assistance. The largest dairy and organic associations (each six in number) were also included. Most of the contacted replied with their willingness to forward the questionnaire. One dairy denied multiplication in a written response.

### Statistical Analysis

The data were extracted from the survey tool and imported into the “IBM SPSS Statistics 27” program (SPSS for Windows, IBM®, Armonk, New York, USA) for further analysis.

Out of 434 questionnaires, 166 were fully completed, 268 were partially completed, and 216 were analyzed. All questionnaires that were completed at least up to page three, that included all questions of general farm data, available labor force and relative importance of subjective VHHM definition, a question on animal health decision making as well as satisfaction with veterinarian were included in the analysis. These questionnaires were examined for duplication using the SPSS function and then subjected to further plausibility checks. No duplications were identified, missing values were not filled, and implausible values were excluded but not replaced. Frequency tables were created for categorical variables. Continuous variables were assessed for the normality of distribution using histograms and boxplots. In order to test the stochastic independence of the variables, the Chi-Square test, the Fisher's Exact test and the Wilcoxon Rank test were performed in the part of descriptive farm data.

The mean values of the variables “advantages,” “disadvantages,” “fulfillment of expectations by a veterinarian,” “cooperation with a veterinarian,” and “improvements of VHHM” (matrix questions with Likert scale) were used to calculate the overall satisfaction with the current VHHM for each participant.

Furthermore, to determine the scope of a farm's VHHM program, each VHHM component was scored based on its sub-questions (e.g., VHHM component “Udder health” included the sub-questions “evaluation of herd performance data,” “milk sampling,” “assessment of parlor routine”). Each component was weighted equally, and the weight of the individual sub-questions was adjusted according to the number of sub-questions. Agreement on all sub-questions in all VHHM components would have resulted in a scope of 100%.

The correlation coefficient was used to determine the undirected correlations between two continuous variables. Thus, the rank correlation coefficient according to Spearman makes it clear in which direction and in which intensity a certain correlation exists. For this, the variables must be at least ordinally scaled (38, 39). To determine the Bravais/Pearson's correlation coefficient, the variables under consideration must be metrically scaled and normally distributed (38, 39). If both variables were normally distributed, the Pearson's correlation coefficient was calculated; otherwise, the Spearman's correlation coefficient was calculated.

Within the non-VHHM farms, we investigated whether the participants could be grouped according to their personal views. For this purpose, a latent class analysis (LCA) was used, which was calculated using the program “SAS Version 9.4” (SAS Institute Inc., Cary, NC, USA). An LCA serves as a statistical model for the exploratory analysis of a dataset. The observed, that is, manifest, variables are checked for unobserved (i.e., latent) correlations to determine the so-called traits. This allows observations to be categorized into two or more groups based on latent classes. The responses of non-VHHM farms were assessed using several possible classes. The subjectively perceived need for VHHM, satisfaction with the veterinarian, herd size, willingness to pay for VHHM, and the presence of other non-veterinary consultants were taken into consideration in the formation of classes. Models with two, three, and four classes were calculated for the four and five variables mentioned, respectively, and compared in terms of interpretability and fit statistics like Akaike information criterion (AIC) (40). Both item-response probabilities and class prevalence were used to assess the interpretability and characterize the classes. The model with two classes and four variables had the lowest AIC and was found to be useful. The variable “presence of non-veterinary consultants” was originally taken into consideration, due to the assumption of a disproportionate presence of the said on the non-VHHM farms and in order to assess the replacement of possible veterinary tasks by the aforementioned.

The free text field on “Motivation for VHHM participation” was analyzed based on Mayring's qualitative content analysis using the independent six-eye principle (41). This analysis technique is used to evaluate the response material by abstracting the individual content to manageable supergroups. The message of the original material is preserved in this process. The advantage of this open approach is the unbiased analysis of individual responses with a simultaneous reduction in scope. The individual steps suggested by Mayring were carried out analogously, and the material was the free text response in the present survey with the above origin and motivation. The answers were analyzed inductively and structured. A coding system was then created, which was then used to review the response material in several sections. After the first author had applied this procedure, two other persons, independent of the project and partly independent of the subject, were assigned this task. Intermediate results were not exchanged at any time, so that the first author only compared all three analyses results at the end of the survey. This independent six-eye principle helps ensure objectivity in the interpretation of free-text responses (41).

For satisfaction with accounting, we determined the proportion of participants who provided the same response on the questions “current billing method” and “desired billing method.” When answers given on the two questions were equal, we concluded that the person is satisfied with the current method of accounting.

## Results

### Participants vs. Non-participants in VHHM

As shown in the descriptive statistics in Table 1, half of the respondents (*n* = 106) responded that they participated in VHHM, while 110 participants responded that they did not participate in VHHM. In both groups, half of the participants belonged to the 30–49-year age group. Three-quarters of the respondents were the respective farm manager, while the remaining respondents were family members or leading employees. The North, East, South, and West regions of Germany were represented by a quarter of each of the non-VHHM farms; among the VHHM farms, those in the East (8.3%) and South (11.1%) regions had low participation rates. Less than 7% of VHHM farms reported farming organically, while 16.4% of non-VHHM farms reported farming organically (Table 1).

**Table 1:** Farm demographics and animal husbandry.

		**Participation in VHHM**			
***(Total: 216)***		**Yes**		**No**	
	*p*-value	*n*	%	*n*	%
	(p_F_ = Fisher's exact; p_C_ = Chi Square)	106	49.1%	110	50.9%
**Age**	*p_*F*_ 0.0914*	*n = 72*		*n = 100*	
<30 years		12	16.7%	25	25.0%
30–49 years		35	48.6%	54	54.0%
50–65 years		23	31.9%	21	21.0%
>65 years		2	2.8%	0	0.0%
**Region**	*p_*F*_ 0.0226*	*n = 72*		*n = 101*	
North		28	38.9%	27	26.7%
East		6	8.3%	19	18.8%
South		8	11.1%	23	22.8%
West		30	41.7%	32	31.7%
**Position**	*p_*F*_ 0.8776*	*n = 72*		*n = 101*	
Farm manager		56	77.8%	75	74.3%
Successor		8	11.1%	15	14.9%
Herdsman		6	8.3%	9	8.9%
Other		2	2.8%	2	2.0%
**Form of cultivation**	*p_*C*_ 0.0250*	*n = 106*		*n = 110*	
Conventional		99	93.4%	92	83.6%
Organic		7	6.6%	18	16.4%
**Form of husbandry**	*p_*F*_ 0.0323*	*n = 106*		*n = 110*	
Free stall—without access to exercise area		42	39.6%	49	44.5%
Free stall—with access to exercise area		28	26.4%	12	10.9%
Free stall—with access to pasture		33	31.1%	43	39.1%
Tie stall—without access to exercise area		0	0.0%	1	0.9%
Tie stall—access to pasture		3	2.8%	5	4.5%
**Form of stalls**	*p_*F*_ 0.1558*	*n = 104*		*n = 104*	
Raised stall—with mattress		44	42.3%	48	46.2%
Raised stall—without mattress		4	3.8%	11	10.6%
Deep bedded cubicle		53	51.0%	42	40.4%
Bedded pack		3	2.9%	3	2.9%
**Usage of AMS**	*p_*C*_ 0.3521*	*n = 106*		*n = 110*	
yes		32	30.2%	27	24.5%
no		74	69.8%	83	75.5%
**Future plan in 10 years**	*p_*F*_ 0.2692*	*n = 72*		*n = 101*	
Continue farm as usual		26	36.1%	32	31.7%
Expand number of milking cows		16	22.2%	19	18.8%
Reduce number of milking cows		2	2.8%	2	2.0%
Hand over the farm to a successor		16	22.2%	15	14.9%
Give up the dairy farm		1	1.4%	5	5.0%
Restructure the farm differently		3	4.2%	13	12.9%
I do not know		8	11.1%	12	11.9%
Others		0	0.0%	3	3.0%
**Open house day**	*p_*C*_ 0.6026*	*n = 72*		*n = 101*	
Yes		45	62.5%	67	66.3%
No		27	37.5%	34	33.7%
**Highest qualification of staff**		*n = 105*		*n = 107*	
Without experience		5	4.8%	4	3.7%
>5 years of relevant experience		11	10.5%	10	9.3%
Apprentice		7	6.7%	10	9.3%
Dairy herdsman		1	1.0%	0	0.0%
Trained farmer (degree)		81	77.1%	83	77.6%

A slight difference was observed between the two subgroups in terms of the type of housing and bed type: animals were kept in free stall barns, while the animals were tethered in only 2.8% of VHHM farms and 5.4% of non-VHHM farms. 51.0% of VHHM farms and 40.4% of non-VHHM farms kept animals in a free stall with deep-bedded stalls, while 46.1% of VHHM farms and 56.8% of non-VHHM farms used a free stall with high stalls (Table 1). 30.2% of VHHM farms reported using an AMS, while only 24.4% of non-VHHM farms used an AMS.

When asked about the plans in the next 10 years, 36.1% of the VHHM farm owners responded that they planned to continue the operation, while only 31.7% of the non-VHHM farm owners wanted to continue the operation. Moreover, 12.9% of the non-VHHM farm owners responded that they wanted to restructure the farm (e.g., start organic milk production), while only 4.2% of VHHM farm owners responded that they wanted to do so.

There was no difference when asked about their willingness to host an open house day where interested citizens could visit the farm: two-thirds in both groups agreed that they would be willing to offer an open house to the public. In both groups, the highest vocational qualification on more than three-quarters of the farms was a trained farmer (degree). The percentage of employees without subject-related experience was <5% in each case (Table 1).

As regards farm data, differences were observed in the two groups (Table 2). The VHHM farms had a mean total number of animals (total stock kept for milk production, including young stock) of 491 with an average milk yield of 22.58 kg ECM (energy corrected milk) per day, while the non-VHHM farms had a mean herd size of 360 animals with 20.20 kg ECM per day on average. The average AFC values were 26 months for VHHM farms and 1 month more for non-VHHM farms. By contrast, the differences in other key performance indicators were less pronounced; for example, the BTSCCs of two MLP-measurements in the last 2 months were 176,000 cells/ml of milk in VHHM farms and 179,000 cells/ml of milk in non-VHHM farms. The RR values were 28% for the VHHM farms and 27% for the non-VHHM farms. On average, 94 animals (total stock for milk production, including young stock) were cared for by each staff member in VHHM farms, while 84 animals were cared by each staff member in non-VHHM farms.

**Table 2:** Farm characteristics.

		**VHHM participation**	
		**Yes**	**No**
**Total number of animals for milk production** (incl. offspring)	*n*	106	110
	25%	150	120
	Mean	491	360
	Median	243	200
	75%	479	400
	SD	978	450
	*p*-value		0.0793
**Number of animals: lactating/dry**	*n*	106	110
	25%	76	65
	Mean	217	191
	Median	130	105
	75%	270	238
	SD	225	228
	*p*-value		0.0869
**305-day milk yield** in kg	*n*	106	110
	25%	9,500	8,000
	Mean	10,195	8,977
	Median	10,399	9,120
	75%	11,200	10,100
	SD	1,524	1,793
	*p*-value		<0.0001
**Energy corrected milk** in kg	*n*	105	107
	25%	21.18	17.73
	Mean	22.58	20.20
	Median	23.09	21.05
	75%	24.56	23.13
	SD	2.93	4.16
	*p*-value		<0.0001
**Bulk tank somatic cell count** in thousands/ml (average of last 2 months)	*n*	106	110
	25%	125.50	130.50
	Mean	176.23	179.16
	Median	165.50	178.25
	75%	226.00	224.00
	SD	69.47	78.06
	*p*-value		0.5434
**Age at first calving** in months	*n*	106	110
	25%	25	25
	Mean	26	27
	Median	26	26
	75%	27	28
	SD	2	3
	*p*-value		0.0020
**Replacement rate** in %	*n*	82	76
	25%	23	20
	Mean	28	27
	Median	28	28
	75%	32	34
	SD	6	9
	*p*-value		0.9763
**Mortality** **<** **60 days in milk**	*n*	59	56
	25%	1.00	0
	Mean	5.47	4.89
	Median	2.00	2.50
	75%	8.00	5.00
	SD	6.82	6.57
	*p*-value		0.2527
**Staffing ratio: total stock** (# animals/staff)	*n*	106	110
	25%	54.59	48.00
	Mean	93.65	84.19
	Median	80.24	72.86
	75%	100.00	105.00
	SD	82.24	67.05
	*p*-value		0.3507
**Staffing ratio: lactating/dry** (# animals/staff)	*n*	106	110
	25%	30.00	26.00
	Mean	48.05	45.13
	Median	43.07	38.13
	75%	54.67	55.96
	SD	35.80	36.42
	*p*-value		0.3401

Table 3 shows that more than three-quarters of VHHM farms “always” or “often” discussed animal health-related decisions with their veterinarian beforehand, while this was only the case in 57.2% of non-VHHM farms. 3.8% of the VHHM dairies “rarely” or “never” made use of advice from their veterinarians, while this was the case of <15% of non-VHHM farms. When asked about general satisfaction with the work of the farm veterinarian, 37.7% of VHHM farms and 28.2% of non-VHHM farms replied “very good.” Combined answers of “very good” and “good” resulted in a value of 80.2% for VHHM farms and a value of 77.3% for non-VHHM farms. “Poor” and “insufficient” satisfaction ratings were awarded by 6.6% of VHHM farms and 8.1% of non-VHHM farms.

**Table 3:** Decision-making and satisfaction with veterinarian.

**VHHM participation**	**Yes**		**No**	
**Decision-making with veterinarian**	*n* = 106		*n* = 110	
Always	21	19.8%	15	13.6 %
Often	63	59.4%	48	43.6%
Occasionally	18	17.0%	31	28.2%
Rare	2	1.9%	15	13.6%
Never	2	1.9%	1	0.9%
**Satisfaction with veterinarian**	*n* = 106		*n* = 110	
Very good	40	37.7%	31	28.2%
Good	45	42.5%	54	49.1%
Satisfactory	14	13.2%	16	14.5%
Sufficient	4	3.8%	5	4.5%
Poor	3	2.8%	4	3.6%

A ranking question was used, to assess the participants' subjective definition of the term “Veterinary Herd Health Management,” through which the participants were asked about their relative importance. Most of the participants ranked the answer “pregnancy checks/advice on reproduction” as number one (Table 4). For VHHM farms and non-VHHM farms, 50 and 40% of the participants provided the same ranking, respectively. Moreover, both groups assigned the answer “improvement of farm management/cost-benefit analyses” the lowest rank.

**Table 4:** Subjective definition of VHHM. Relative importance of subjective VHHM definition. *(VHHM: 106; non-VHHM: 110)*.

		**Rank 1**	**Rank 2**	**Rank 3**	**Rank 4**	**Rank 5**
“Pregnancy checks/consultation on reproduction”	VHHM	50.0%	24.6%	10.1%	7.6%	7.6%
	Non-VHHM	40.0%	25.5%	9.9%	10.9%	13.6%
“Discussion of herd production data”	VHHM	0.9%	16.0%	27.4%	36.8%	18.9%
	Non-VHHM	3.6%	14.5%	26.4%	33.6%	21.8%
“Tour through all stages of production/strategy discussion”	VHHM	14.2%	13.2%	26.4%	29.2%	17.0%
	Non-VHHM	13.6%	12.7%	33.6%	24.5%	15.5%
“Identifying and addressing current herd health problems”	VHHM	31.1%	39.6%	18.9%	8.5%	1.9%
	Non-VHHM	40.9%	40.9%	11.8%	3.6%	2.7%
“Improving farm management/cost-benefit analyses”	VHHM	3.8%	6.6%	17.0%	17.9%	54.7%
	Non-VHHM	1.8%	6.4%	18.2%	27.3%	46.3%

### Participants in VHHM

As shown in Table 5, Mayring's content analysis indicated that the main motivation for participation was to ensure animal health on the farm, since “animal health/animal welfare (prophylaxis)” found most approval. This also crystallized in the ranking question, in which “remedying herd health problems” was assigned a high importance. Increasing milk yield played a subordinate role, and participation due to external regulations, such as obligations by the dairy farm, was provided the lowest priority.

**Table 5:** VHHM: Motivation of participation.

**Motivation to participate in VHHM (free definition) *(n* = *94)***	***n***	**%**
“Animal health/animal welfare (prophylaxis)”	40	42.55
“Optimizing performance/efficiency”	21	22.34
“Reproduction”	16	17.02
“General help/management/problem identification/broadening of horizon”	35	37.23
“Protocols (e.g., through dairy/slaughter plant)”	8	8.51
**Motivation to participate in VHHM (ranking)** *(n* = *97)* [1 = fully applicable −5 = not applicable at all]		Mean
Remedy herd health problems		1.97
Prevention of operational blindness		2.00
Avoid conflicts of law		2.10
Profit optimization		2.38
Control of production data		2.44
Recommended by veterinarian		2.71
Work structuring/sharing administration work with veterinarian		2.78
Required by higher authority		3.92

Potential advantages of VHHM were rated similar on the Likert scale (1 = fully applicable, 5 = not applicable at all), with “More timely problem detection” (mean: 2.04) and “better herd health” (mean: 2.08) having had the lowest mean values. The disadvantages met with less approval. Here, “High costs” (mean: 3.04) and “very time-consuming” (mean: 3.15) had the lowest mean values (Table 6).

**Table 6:** VHHM: Perceived advantages/disadvantages and fulfillment of expectations.

**Advantages of VHHM and ranking *(n = 98)* [1 = fully applicable −5 = not applicable at all]**		**Mean**	**SD**
More timely problem identification		2.04	0.849
Better herd health		2.08	0.833
Prevention of operational blindness		2.21	0.853
Better farm management		2.22	0.844
More structured problem-solving		2.32	0.892
Better herd performance		2.45	0.863
Organization has improved		2.46	0.943
Information on subject-related development		2.46	0.954
Control of production data		2.64	0.997
**Disadvantages of VHHM and ranking** *(n* = *98)* [1 = fully applicable −5 = not applicable at all]			
High costs		3.04	0.962
Very time-consuming		3.15	0.956
Difficulties with data collection		3.64	0.977
Non-tailored advice		3.85	0.945
Inappropriate visiting hours		3.95	0.988
Little experience of veterinarian/not enough good advice		3.95	1.170
Advice not useful		3.97	0.779
Veterinarian interferes too much in management		4.07	0.750
**Fulfillment of the expectation in VHHM** *(n* = *90)* [1 =very good −5 = insufficient]	***n***	**Mean**	**SD**
Fertility	84	1.67	0.781
Animal welfare	43	1.79	0.742
Claw health	46	1.98	0.856
Young stock health	45	1.98	0.917
Udder health	75	1.99	0.893
Biosecurity	22	2.00	0.873
Facilities/animal husbandry	15	2.40	1.121
Staff management/education	8	2.13	0.991
Performance/herd data	35	2.14	0.879
Nutrition	36	2.28	1.059
Farm economics	7	2.71	0.488
**If some advice does not have a desired outcome, what might be the reason?** *(n* = *90)*		*n*	%
I followed the advice, but it failed because…	…the advice did not correctly address the cause of the problem.	30	33.3
	… the correct cause was addressed and implemented but still no effect occurred.	42	46.7
I did *not* follow the advice because…	… the advice was not practicable in everyday life.	11	12.2
	… the advice did not seem useful to me.	7	7.8

The area of herd fertility management was the most frequently assessed part in VHHM; from the participants' point of view, the veterinarian fulfilled the expectations placed on him or her, particularly in this field. Farm economics, on the other hand, was only very rarely (8%) part of the VHHM, and the role of the veterinarian in this regard was rated as comparatively less satisfactory (mean = 2.71) (Table 6).

Also evident from Table 6 are results of why veterinary recommendations did not always lead to the desired outcome. 46.7% of the participants responded that they implemented the veterinarian's advice, and the cause was also correctly addressed, but the problem could not still be resolved for other reasons. Meanwhile, 7.8% of the participants did not follow the advice because they considered it impractical.

**Figure 1:**
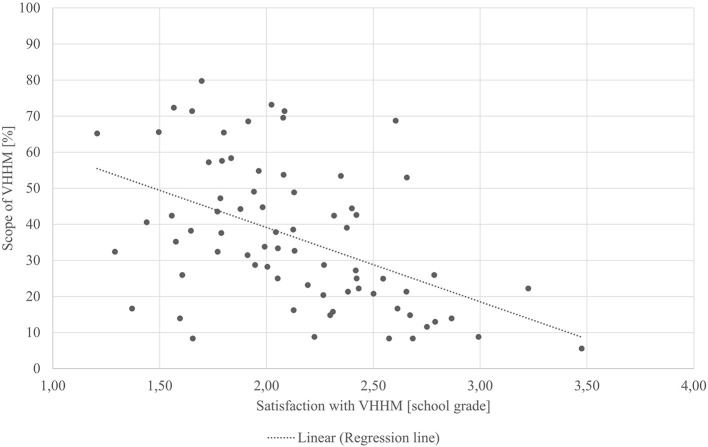
Scatterplot: Scope/satisfaction with VHHM.

As Table 7 shows, the calculated satisfaction rate with the current VHHM was normally distributed and had a mean value of 2.18 (“good”). This satisfaction rate correlated significantly negatively with the VHHM scope, that is, the utilized proportion of all areas that the VHHM could possibly cover (r_P_ = −0.477, *p* < 0.001); as shown in Figure 1, the higher the scope of VHHM, the better the average satisfaction rate. Decision-making with the veterinarian was similarly correlated with VHHM satisfaction (r = −0.402, *p* < 0.001). The more satisfied participants were, the more often they made health-related decisions together with their veterinarians. VHHM satisfaction was positively and significantly correlated with the general satisfaction with the veterinarian (r = 0.576, *p* < 0.001). When VHHM appointments were scheduled independently of the veterinarian's curative visits, this increased the animal owners' satisfaction with the VHHM (r_S_ = 0.367, *p* < 0.001). Animal owners who were satisfied with the VHHM also perceived greater financial value (r_S_ = 0.563, *p* < 0.001). These participants would continue VHHM even if the fee for this service increased by 10% (r_S_ = 0.266, *p* = 0.021).

**Table 7:** VHHM: Satisfaction with VHHM/scope of VHHM and importance of VHHM subjects.

**Satisfaction with VHHM** [1 = very good −5 = insufficient]							
***n***	**25%**	**Mean**	**Median**	**75%**	**SD**	**Shapiro wilk**	
						**Statistics**	**Sig**.
98	1.80	2.18	2.13	2.42	0.48	0.97	0.013
** Correlation**					*n*	r (r_P = Pearson;_ r_S = Spearman−Rho_)	*p*-value
Scope of VHHM [0–100%]					73	r_P_ −0.477	<0.001
Satisfaction with veterinarian [1 = very good −5 = insufficient]					98	r_S_ 0.576	<0.001
Decision making with veterinarian [1 = never −5 = always]					98	r_S_ −0.402	<0.001
Herd visit (in-)dependent of curative visit [1 = yes/2 = no]					98	r_S_ 0.367	<0.001
Financial added value [1 = fully applicable −5 = not applicable at all]					85	r_S_ 0.563	<0.001
Participation if VHHM fee is increased by 10% [1 = yes; 2 = yes, but reduced hours; 3 = no]					75	r_S_ 0.266	0.021
**Scope of VHHM** [0–100%]							
**n**	**25%**	**Mean**	**Median**	**75%**	**SD**	**Shapiro wilk**	
						**Statistics**	**Sig**.
68	21.02%	36.41%	33.33%	51.02%	19.76%	0.953	0.008
**Correlation**					n	r (r_P = Pearson;_ r_S = Spearman−Rho_)	*p*-value
Satisfaction with veterinarian [1 = very good −5 = insufficient]					73	r_S_ −0.320	0.006
Decision-making with veterinarian [1 = never −5 = always]					73	r_S_ 0.366	0.002
Herd visit (in-)dependent of curative visit [1 = yes/2 = no]					73	r_S_ −0.363	0.002
Recording of the current state and setting goals [1 = yes/2 = no]					73	r_S_ −0.583	<0.001
Setting written goals [1 = yes/2 = no]					73	r_S_ −0.369	0.001
Establishing a cost-benefit analyses [1 = yes/2 = no]					73	r_S_ 0.494	<0.001
Financial added value [1 = fully applicable −5 = not applicable at all]					73	r_S_ −0.416	<0.001
Participation if VHHM fee is increased by 10% [1 = yes; 2 = yes, but reduced hours; 3 = no]					73	r_S_ −0.232	0.049
Farm size (number of animals lactating/dry)					73	r_P_ 0.051	0.671
**Ranking: Importance of VHHM subjects** (*n* = 73)						**Rank**	**Mean**
Fertility						1	1.40
Udder health						2	1.81
Claw health						3	2.79
Young stock health						4	2.97
Animal welfare						5	3.03
Nutrition						6	3.07
Herd data						7	3.33
Biosecurity						8	3.56
Farm economics						9	3.78
Facilities						10	3.90
Staff management/training						11	4.05

**Figure 2:**
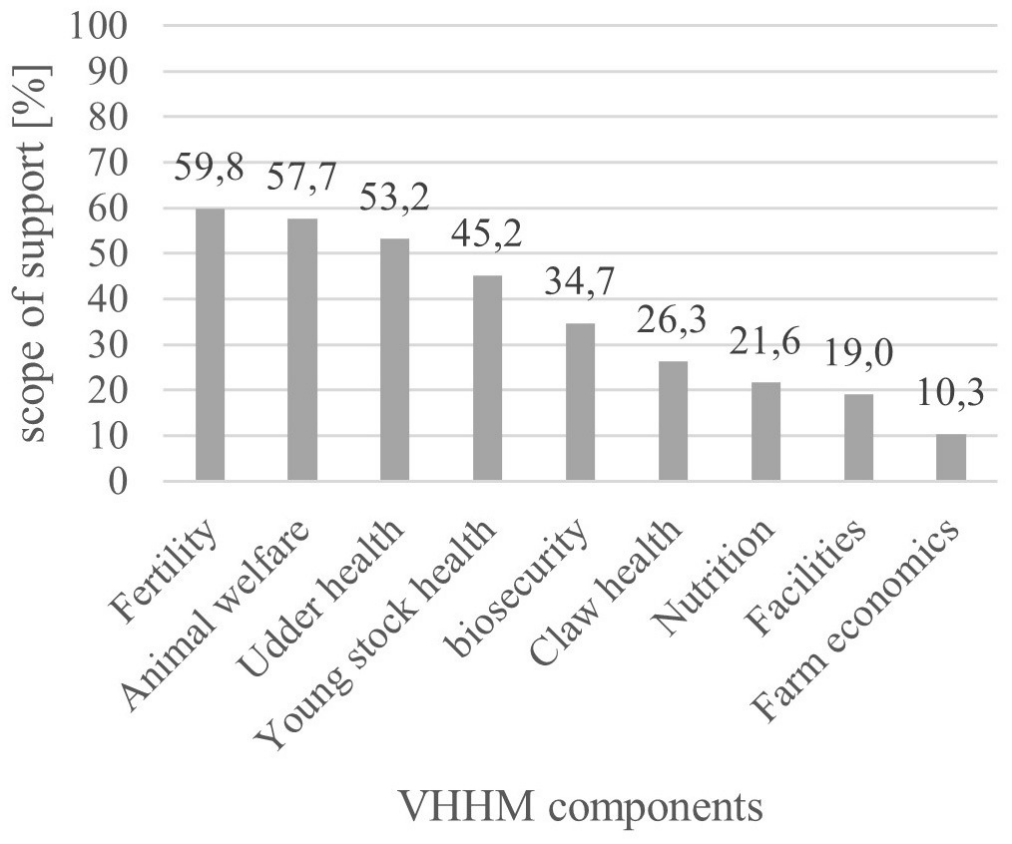
Bar chart: Scope of VHHM subjects.

The average scope of VHHM was 36.41% and was normally distributed (Table 7). The most intensively attended areas were fertility (59.8%) and animal welfare (57.7%), while farm economics (10.3%) was the least intensively attended area (Figure 2).

Table 7 shows additionally, that the more intensively a farm was attended, the more satisfied its farm manager was with the veterinarian as a person (r_S_ = −0.320, *p* = 0.006). Moreover, the more intense dairy farms were supported, the higher the probability of making decisions together with the veterinarian (r_S_ = 0.366, *p* = 0.001). Support was equally more intensive when the visit was independent of curative veterinary visits (r_S_ = −0.363, *p* = 0.002). Moreover, the scope of support correlated with the recording of current status (r_S_ = −0.583, *p* < 0.001), writing down goals (r_S_ = −0.369, *p* = 0.001), and use of cost-benefit analyses (r_S_ = −0.494, *p* < 0.001). The more intensive the support, the more likely the previously mentioned aspects were. Likewise, there was a significant correlation between financial added value and support scope (r = −0.416, *p* < 0.001).

The personal ranking of importance in the fields worked on during VHHM visits was led by reproduction (mean = 1.40) and closely followed by udder health (mean = 1.81). Staff management/training was evaluated as an area of low relevance (mean = 4.05) (Table 7).

Table 8 clarifies that nearly two-thirds (59.0%) of the veterinarians were satisfied with their current method of accounting for services provided as part of the VHHM program. Charging by the hourly rate for services provided as part of the veterinarians' consultation and practical work was preferred (42.9%). Only 6.7% of the farmers would terminate VHHM services if the associated costs increased by 10% or more. Almost two-thirds of the participants would continue in the usual way under the previously mentioned conditions. The remainder would continue to use the VHHM service but reduce the number of hours. More than half of the participants indicated that their veterinarians' pre- and post-procedure hours were not billed. Only 21.2% of the respondents accounted for this time. When asked if VHHM generated added financial value on the farm, there was an average of agreement (“agree;” mean = 2.14).

**Table 8:** VHHM: Accounting method.

**Satisfaction with current accounting method *(n = 83)***			***n***	**%**
Overall satisfied			49	59.0%
Hourly rate (including all advisory and practical services performed)			47	56.6%
Hourly rate (practical services are charged extra)			16	19.3%
Fixed rate per animal and year			7	8.4%
Module form			5	6.0%
Total pack/flat rate for farm			7	8.4%
Success fee			1	1.2%
**Desired accounting method** *(n = 84)*			n	%
Hourly rate (including all advisory and practical services performed)			36	42.9%
Hourly rate (practical services are charged extra)			15	17.9%
Fixed rate per animal and year			6	7.1%
Module form			3	3.6%
Total pack/flat rate for farm			14	16.7%
Success fee			10	11.9%
**Further participation if VHHM fee is increase by 10%** *(n = 75)*			n	%
No			5	6.7
Yes	With reduced number of hours		22	29.3
	With same number of hours		48	64.0
**Accounting for veterinarian's preparation and follow-up time** *(n = 85)*			n	%
No, not accounted for			48	56.5
Yes, accounted for:	Separately		12	14.1
	Not separately		6	7.1
Unknown			19	22.4
**Financial added value through VHHM**		*(n = 48)*		Mean = 2.14

**Figure 3:**
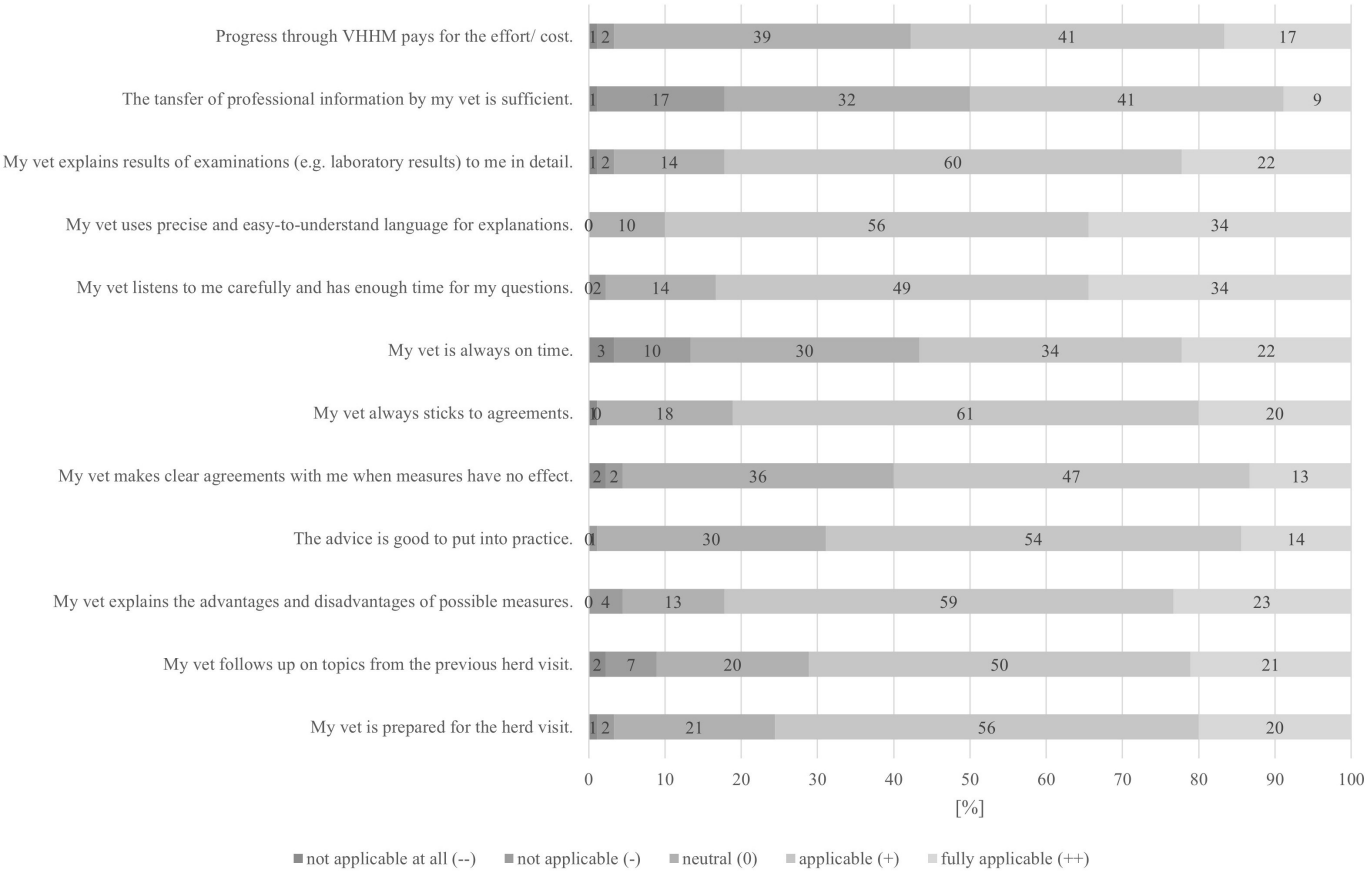
Bar chart: Cooperation between farmer and veterinarian.

Figure 3 shows that the participants in the survey placed a high value on the fact that explanations were given in an understandable language, as well as had the ability to listen actively and to spend enough time to answer questions. The fact that progress pays off in terms of effort and cost was provided an average rating of 2.14.

**Figure 4:**
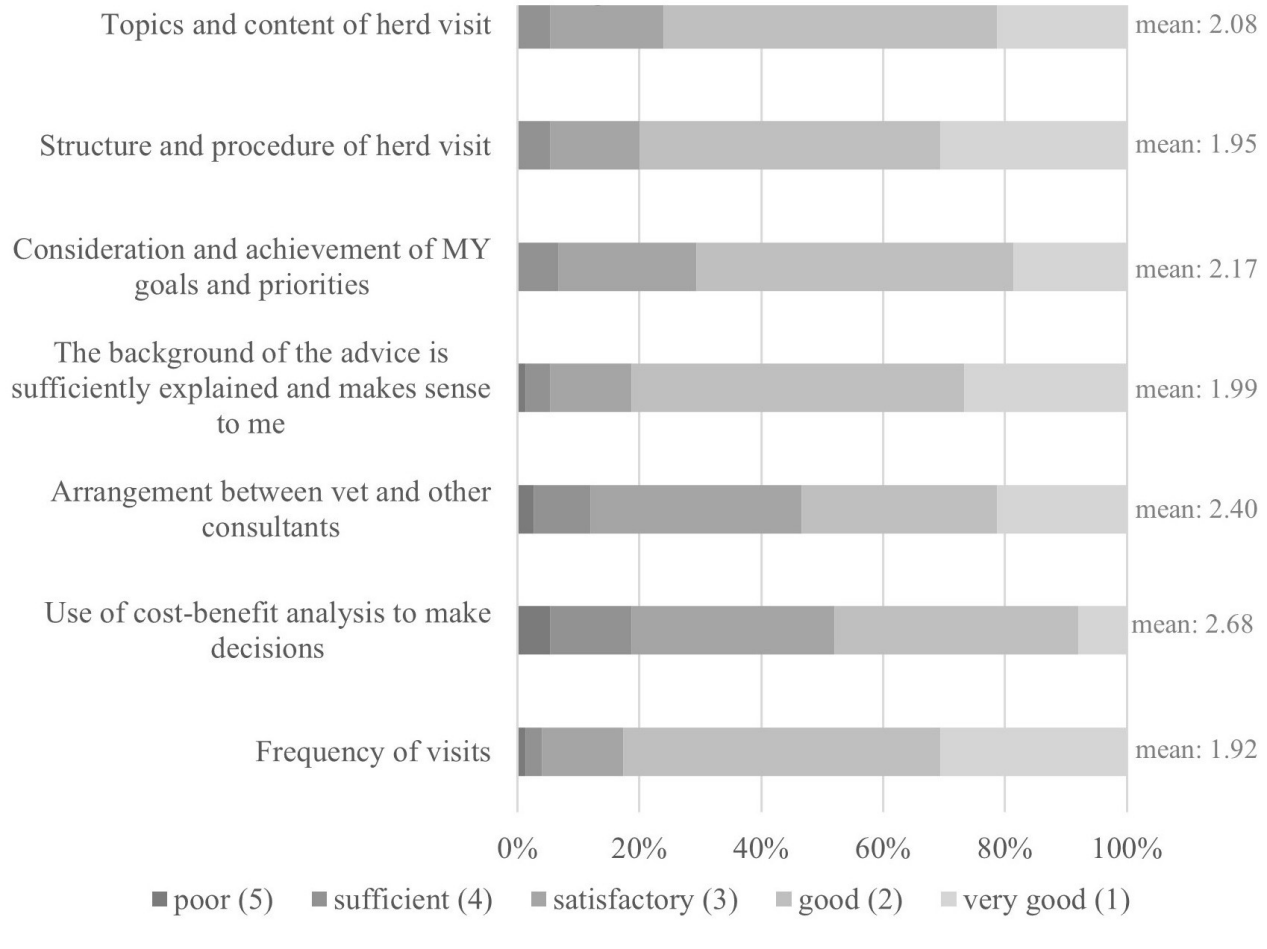
Bar chart: Quality of current VHHM.

In order to outline the current VHHM quality on the farms (Figure 4), the participants were asked about the potential for improvement of the following aspects: topics, content and structure of a herd visit, consideration of farm-tailored goals, frequency of visits, use of cost-benefit analyses, consultation with other advisors, and comprehensibility of advice. The lowest mean value of these school grade ratings, and thus the highest consent, was “frequency of visits” (mean: 1.92) and the highest mean value, equaling lowest consent, was assigned to the use of cost-benefit-analyses for decision-making (mean: 2.68).

### Non-participants in VHHM

Only four participants had used VHHM in the past but later abandoned it because they were either dissatisfied with the outcome, found the tariff too high, or considered the high time commitment unreasonable. The fourth participant cited a “change of veterinarian” as the reason for quitting VHHM.

Only 12.7% of non-VHHM farms were convinced that they do not need VHHM on their farms (Table 9). Just over half were unsure, and one-third said they had a need but were not currently receiving herd health management services. The areas in which participants saw the greatest or highest need were hoof health, fertility, and udder health.

**Table 9:** No VHHM participation: Descriptive data.

**Previous participation in VHHM (*n* = 106)**				***n***		***%***
Yes				4		3.8%
No				102		96.2%
**Need for VHHM?** (*n* = 102)				*n*		%
Yes				36		35.3%
Unsure				53		52.0%
No				13		12.7%
**… if “yes”/”unsure”: conceivable need in …?** (*n* = 8		**Yes**			**No**	
	*n*		%	*n*		%
fertility	46		51.7%	43		48.3%
Udder health	45		50.6%	44		49.4%
Performance/evaluation of herd data/digitalization	25		28.1%	64		71.9%
Claw health	51		57.3%	38		42.7%
Young stock health	18		20.2%	71		79.8%
Nutrition	29		32.6%	60		67.4%
Facilities/animal husbandry	15		16.9%	74		83.1%
Biosecurity	18		20.2%	71		79.8%
Farm economics	17		19.1%	72		80.9%
Animal welfare	19		21.3%	70		78.7%
Staff management/training	13		14.6%	76		85.4%
**Willingness to pay minimum hourly rate according to GOT** (*n* = 106)	*n*		%			
Yes	35		33.0%			
No	71		67.0%			
**Number of consulting veterinary practices** (*n* = 106)	*n*		%			
1	95		89.6%			
2	11		10.4%			
**Performance of above-mentioned additional practices** (*n* = 11)				**Yes**		**No**
	*n*		%	*n*		%
Fertility	8		72.7%	3		27.3%
Drug purchase	9		81.8%	2		18.2%
Nutritional advice	1		9.1%	10		90.9%
**Non-veterinary advisors** (*n* = 106)		**Yes**		**No**	
	*n*		%	*n*		%
	76		71.7%	30		28.3%
**Nature of the abovementioned non-veterinary advice** (*n* = 76)						
Nutritional advice	67		88.2%	9		11.8%
AI technician/cattle breeder association	47		61.8%	29		38.2%
Agricultural advisor	16		21.1%	60		78.9%
Animal health service	11		14.5%	65		85.5%
Dairy/department of quality management	25		32.9%	51		67.1%
Regional advisory board	25		32.9%	51		67.1%

Two-thirds of the participating farms were not willing to pay the minimum hourly rate of *$*89.32 for veterinary consultation required by the German Scale of Veterinary Fees (GOT).

The majority (89.6%) of the farms were only served by one veterinary practice. Other veterinarians served the remaining farms mainly for support in herd fertility management (8 out of 11) and medication purchases (9 out of 11).

Non-veterinary consultants were present in 71.7% of the farms surveyed and were consulted mainly for feeding advice (88.2%) or were active as inseminators for the Cattle Breeders Association (61.8%).

With regard to the reasons for not using VHHM, two different “farm types” were identified using latent class analysis (Table 10). The Gamma estimate of belonging to class one was 0.7863, while that of belonging to class two was 0.2137. In class two farms, the associated farmers tended to manage larger farms and at the same time were more likely to be dissatisfied with their current veterinarians. At the same time, it was striking that almost all these farms saw a need for veterinary care and had a significantly higher willingness to pay the GOT minimum hourly rate for this. By contrast, type one farms were smaller farms that did not see the need for VHHM and were also not willing to pay the GOT minimum hourly rate. The main model selection criteria are shown in Table 11.

**Table 10:** No VHHM: Latent class analysis.

**LCA model: 4 variables** **−2 classes** (AIC: 125.02; BIC: 180.14; Adjusted BIC: 113.81; Entropy: 0.73)		
**Class membership probabilities: Gamma estimates (standard errors)**		
Class	1	2
	0.7861 (0.0739)	0.2139 (0.0739)
**Item response probabilities: Rho estimates (standard errors)**		
**Herd Size**		
Small (<70 cows)	0.3025 (0.0550)	0.2186 (0.1152)
Rather small (70–120 cows)	0.2849 (0.0529)	0.1916 (0.1024)
Rather big (121–251 cows)	0.2198 (0.0489)	0.3365 (0.1201)
Big (>251 cows)	0.1928 (0.0479)	0.2533 (0.1137)
**Satisfaction with vet**		
Very good	0.2807 (0.0550)	0.2069 (0.1205)
Good	0.5908 (0.0598)	0.2169 (0.1543)
Satisfactory	0.1137 (0.0389)	0.2678 (0.1155)
Sufficient	0.0145 (0.0154)	0.1737 (0.0934)
Poor	0.0003 (0.0022)	0.1348 (0.0817)
**Possible need of VHHM**		
Yes	0.1864 (0.0718)	0.9549 (0.0792)
Unsure	0.6525 (0.0702)	0.0390 (0.0756)
No	0.1610 (0.0429)	0.0061 (0.0242)
**Willingness to pay the minimum GOT hourly rate**		
Yes	0.2635 (0.0543)	0.6310 (0.1400)
No	0.7365 (0.0543)	0.3690 (0.1400)

**Table 11:** No VHHM: LCA-Model selection based on AIC.

	***4 variables*** **- herd size,** **- satisfaction with vet**, **- possible need of VHHM,** **- willingness to pay**	***5 variables*** **- herd size,** **- satisfaction with vet,** **- possible need of VHHM,** **- willingness to pay,** **- presence of non-veterinary consultants**
2 classes	125	196
3 classes	131	197
4 classes	144	207

A comparison of VHHM-participants with the two LCA classes of non-VHHM participants, is outlined in Table 12. Farmers that do not participate in a VHHM but are interested (LCA class 2) have larger farms than those that do not participate and are not interested (LCA class 1). At the same time, those that do not participate but are interested in a participation (LCA class 2) are more dissatisfied with the current farm veterinarian, than the ones participating in VHHM and the ones without interest in participation (LCA class 1).

**Table 12:** Comparison: VHHM-participants and non-VHHM participants with interest in participation.

		**Participants in VHHM (*n* = 106)**	**Non-participants in VHHM**	
			**LCA class 1 (*n* = 86)**	**LCA class 2 (*n* = 24)**
Herd size [# lactating/dry cows]	Mean	217.5	187.3	202.6
	Median	130.0	100.0	178.5
General satisfaction with herd veterinarian [school grade]	Mean	1.92	1.83	2.92
	Median	2.00	2.00	3.00

## Discussion

This study aimed to gain insight into the status quo of the currently practiced VHHM in Germany since the related study situation in this nation is scarce. Half of the study participants took part in the VHHM.

According to the Federal Statistical Office of Germany, 57,322 dairy farms in the entire country have already been registered in 2020 (42). Thus, the participation of 216 farms represented 0.38% of the population of dairy farms. Questionnaire dropouts were reviewed and showed slightly more VHHM participants who did not complete the questionnaire. The reason for this could be the more time-consuming questionnaire for this group of participants. The amount of fully completed questionnaires were divided about half each into VHHM-participants and non-VHHM-participants.

The fact that the survey was exclusively accessible online provides a reason to believe that selection bias was present. To avoid further bias, the survey was intentionally not advertised during veterinary visits to avoid weighting the specific practices of VHHM. However, online recruitment only targeted dairy farms with email addresses and membership in association mailing lists or access to social media. An equally large factor was the participant's personal affinity for online media and their own motivation for the relevant topic (43). There was also a discrepancy between the mean number of lactating and dry cows per farm in our study and the 2020 nationwide average of dairy and dry cows (42, 44). This is not unexpected, as a previous study also showed a change in study participants, from smaller farms to larger farms (30). This can be explained by the fact that larger farms tend to be more proactive and, thus, more likely to show interest in current topics and to conduct surveys related to these topics (45). Consequently, this fact could lead to an overestimation of the proportion of VHHM program participants. In order to make a representative statement about the target group, a larger-scale follow-up study would be one way to get to the bottom of this research question. The study undoubtedly contains a certain bias, also due to the relatively extensive questionnaire. Therefore, the representativeness for Germany must be evaluated especially against this background. The transferability to other countries probably behaves in such a way that the type of dairy farming should be similar, and this depends on the respective farm structure. Nevertheless, Germany, as one of the leading dairy farming nations worldwide, offers a good cross-section in this field, as the country is very diverse in terms of farm structure. With this in mind, it can be assumed that the results of this study provide a good point of reference, especially for northern and western European countries, where similar studies were conducted before (13–18, 46). With regard to the legal basis, all EU member states are now facing the immediate implementation of the animal health law. Regarding the development over time, it is more of an outlook than a retrospective, as the latest change in the law is likely to be structurally significant.

Participation in the survey was explicitly voluntary and anonymous, but the results must nevertheless be interpreted against the background that participants tend to give a distorted picture of themselves (47). Specially in the case of the queried performance parameters, there could be a deviation from reality, as the farm might have been portrayed better than it is. To prevent this, we indicated in the introduction to prepare the current MLP in advance and provided participants with the exact page and field reference in this document where they would find the required data for the upcoming questions. Owing to this indication and the guaranteed anonymity, we assumed that the information provided was mostly valid.

### Participants vs. Non-participants in VHHM

The participants were deliberately not given a definition of VHHM in the introduction to prevent inhibited participation or supposed misperceptions. However, due to the resulting freedom of interpretation, the participants may have felt that they belonged to the wrong group. This could also explain the high proportion of VHHM farmers whose relative importance was mostly weighted toward regular pregnancy examinations and advice on reproduction, which does not justify the actual idea of a holistic approach to herd management (48). Of the non-VHHM respondents, 40% also interpreted VHHM as a purely reproduction-related service, although an equally large proportion understood it as a means to solve herd problems. Veterinarians should therefore provide targeted and proactive education in order to show the entire spectrum of opportunities that VHHM can offer.

Farms that participated in the program may be positive prospects in the future. VHHM farms were more likely to be willing to expand, while non-VHHM farms were more likely to stop dairy farming. Performance data confirmed this impression, as VHHM farms were larger on average with, for example, higher milk yields and lower AFC, implying a better overall production. This has already been shown in a previous study (26). However, it remains difficult to discern in our study whether VHHM is the cause or effect of this difference.

It is also necessary to differentiate the personnel structure in studied farms: in the literature, the specification of cows per full-time equivalent (FTE) (total number of hours worked by a full-time employee) is common to quantify the efficiency of labor. The cows/FTE ratio has been increasing over the years, possibly because of this labor-intensive enterprise and shortage of workers (49). A study in Michigan, USA, reported a range of 25–105 cows per FTE (50), while a study in California, USA reported a ratio of 82 cows per employee, for smaller farms below 250 head, to 151 cows per employee, for farms with more than 700 cows (51). In our sample, the data were within this range. The staffing ratio, based on the total number of animals in the herd, differed by 10% for VHHM and non-VHHM farms. However, the number of lactating and dry cows per employee was very similar between the two groups. Therefore, in contrast to the study mentioned above, the farm size factor, which would result from the tendency of larger farms to participate in VHHM, seemed to play a minor role in our sample. The variation between the staffing ratio for the total number of animals and that for lactating and dry cows could nevertheless be due to a stronger focus on dairy cows and/or a higher degree of digitization on the VHHM farms. A wider staffing ratio implies better labor efficiency; however, it remains unclear how a larger ratio affects animal welfare and/or performance.

### Participants in VHHM

The result of the two motivation questions for participation in VHHM was congruent: the highest priority for the dairy farmers was the health status of their performing herd. Thus, the pure increase in performance or profit maximization was not given a priority, which is an important signal, especially in view of the critical consumer voices with recurring public discussions as a reflection of the farmers' mindset. The study participants felt primarily committed to maintain the health of their animals; in the long run, this approach also comes to the right conclusion: only a healthy herd and a healthy animal can unfold its potential and perform accordingly. Since the genetic selection for performance may be viewed critically from the perspective of animal welfare (52), maintaining the health of individual animals is a basic element of dairy cow husbandry and is the key aspect of animal welfare (53). Other studies are in agreement with the latter observations, as one study stated that the most important attribute of their survey was the statement of a livestock farmer: “To feel happy knowing that my dairy cows are well-kept” (54). Another study also found, that animal welfare was valued most, compared to pure increase in milk production (16). The results of the ranking question on relative importance of subjective VHHM definition and motivation to participate in a VHHM program contrast with each other to a certain extent: All participants rated “pregnancy checks/consultation on reproduction” highest, while “identifying and addressing current herd health problems” was rated lower, whereas the question about motivation of participation reveals it the other way around. It is possible that then farmers' actual understanding (reflected in the ranking question) is the current practice they experience, while the answer to the question about motivation to participation reflects their theoretical conception or desires of this type of support. Furthermore, it is possible that the motivation of participation for veterinary support puts more focus on (herd) health, because the “health aspect” gives a medical professional a certain monopoly in the minds of farmers, while pregnancy checks or similar could also be performed by other professionals such as AI technicians.

Animal health was also reflected in the subjectively perceived benefits: all participants provided similar ratings to the benefits of VHHM. Early problem detection was identified as the greatest benefit, closely followed by improved herd health. As previously confirmed, farms regarded the threat of operational blindness without external input as an important factor (36). This goes hand in hand with the motivation mentioned above, as VHHM can be valuable because of the external view of the veterinarian. Similar to our results of the motivation and advantage question, a study in the UK found, that the main advantage for participating in VHHM was “Improving health and welfare of the animals” (46). Finally, the veterinarian is one of the most important advisors in a farm (30). In contrast to other studies (36), the perceived disadvantages did not seem to matter much: these were all rated as “neutral” to “does not apply.” As discovered before (17), high costs were still the most important issue, although in our study this cannot be judged as such, due to the weak or neutral ratings. Similar to a previous study (46) the time needed for a VHHM program was also a highly ranked disadvantage. Nevertheless, veterinarians should always address the problems related to the invested costs and, more importantly, show the benefits of making the advantages of VHHM transparent.

As mentioned above, most participants understood that VHHM equals support in “herd reproduction.” This is also reflected in the fulfilled expectations of veterinarians because the area of “fertility” is treated as a priority in the vast majority of farms. For example, the hidden costs of up to 230*$*/cow/cow/year in the case of poor fertility management confirm the importance of the topic (55); nevertheless, future studies may not only focus on VHHM as a single topic. The goal is to approach all production areas of the entire farm. In a publication by a veterinarian from Israel, one of the world's leading countries in dairy farming, the development of production diseases was also described as a consequence of mismanagement due to a ruthless desire to increase performance. VHHM is designed to buffer mismanagement and thus enhance production under optimal regimens (56). In terms of economic impact, hoof health, young stock rearing, and cow comfort (23, 57–59) are areas with high economic impact but are less considered in VHHM. It can be debated whether employee management or farm economics falls within the scope of practice of a veterinarian; nevertheless, it was covered by some practices. In the future, such an offer or cooperation, including experts in this field, could be conceivable.

As mentioned before, the relationship between animal owners and veterinarians is a working relationship of a special kind. Intensive collaboration with a high degree of professionalism under tight economic conditions and sometimes emotionally charged situations, due to the unpredictability of working with living creatures and the high daily workload, characterize this trusting relationship (60). Therefore, it is immensely important that the basis between individuals is correct; otherwise, the construct is not very promising. Researchers from the Netherlands validated this in their study: “Because VHHM is based on preventive advice, the strength of the relationship is an important contributor to the success of VHHM” (26).

The tendency of medical professionals to use medical language when talking to non-medical people does not seem to apply to veterinarians in VHHM. After all, the participants rated their veterinarians best at this point. It is likely, that the participants' agricultural education background and the close relationship and thus, resulting loyalty, constituted a basic satisfaction with the farm veterinarian of the participants in our study.

In their own perceptions, the study's animal owners considered themselves reliable. After all, 80% of the participants said that they followed the advice of the veterinarian. This contrasts with the results of other studies; for example, only 50% of the participants implemented the recommended veterinary measures (12, 19, 33). The answers of our survey and thus, resulting difference with the studies mentioned above may be due to the phenomenon of “socially desirable responding” by participants of a survey (47).

Veterinarians have the potential to influence the quality of the current VHHM. For example, including cost-benefit analyses in their advice, such calculations are useful for any type of new investment in an economically tight field such as dairy farming. Also, not to be neglected is the survey participants' dissatisfaction in the cooperation between veterinarians and other consultants. A collaboration between dairy farmers and all advisors involved is more likely to succeed, than without said communication.

Overall, dairy farmers showed good satisfaction with VHHM, which also coincided with the results of other studies (16, 61). It can be assumed that intensive and long-term collaboration between veterinarians and animal owners has been maintained for years. Consequently, VHHM satisfaction also correlates with VHHM scope. Intensive herd management can keep many aspects overlooked at the same time, and thus a farm is more likely to reach its potential. Furthermore, it is not surprising that decision-making correlated with satisfaction, because a trusting relationship with the veterinarian strengthens regular exchange and thus indirectly regular consultation (29). If decisions must be made, the farmer is happy to consult the veterinarian.

Only the costs of VHHM were viewed critically. As mentioned above, the participants saw the costs as the greatest disadvantage of the VHHM, and their dissatisfaction with the accounting system was correspondingly high. Only <60% of the participants were satisfied with the current form of accounting. This could again be due to the financial difficulties of dairy farming. More than half were accounted for *via* a fixed hourly rate, including all services, the fewest *via* a performance-independent payment. However, the latter would be more desired, possibly to have financial security as a farm and at the same time calculable fixed costs. Here, it would be interesting to determine how high the veterinary costs of individual participants are and whether conclusions can be drawn from it on the (desired) accounting form. In principle, the cost shares of total production for veterinary measures are well-described (62, 63); hence, the conduct of cost-benefit analyses is strongly recommended to show farms the benefits of the invested VHHM costs.

Nevertheless, the participating farms appreciated the added value of VHHM. This was clearly demonstrated by the fact that not even 7% would stop participating despite the increase in the in the VHHM service fee. In 20% of the participants, the time invested by the veterinarian outside of the time for the management on-farm for VHHM was accounted for; this would be a possible approach that can be used in the future in the field of veterinary practice. At least there is recognition from the dairy farms, as they feel it is true that the VHHM offers them added financial value. However, it remains difficult to evaluate the different veterinary consulting activities included in monetary terms, since the indirect economic impact and positive financial impact of disease prevention and improvement in animal welfare is highly complex to monetarize and is additionally of great lag, since it can only be observed at a later time.

The calculated scope of VHHM showed once again that the claim of a holistic approach has not been implemented to a sufficient extent so far and that veterinarians can exploit the existing potential based on the quality they offer. Even if the indicated activities of a VHHM veterinarian do not necessarily involve providing a quality VHHM service, the most important message is that only one-third of the possible VHHM components were included. None of the respondents achieved 100% of the components; however, as mentioned, the bar was also set high. The cause could be insufficient supply on the part of the veterinary profession, but it could be a lack of adoption by farmers given the existing supply. However, further research is needed to examine the specific cause. The scope is strongly correlated with the recording of current status and goal setting. It is recommended to perform a SWOT analysis, agree on goals, write them down, and work toward them (64). Since the scope did not correlate with the size of the farm, these measures are not an attribute of a larger and therefore supposedly well-organized farm; however, a farm of any size can carry out a VHHM of any degree. Notably, the more intensively a farm was managed, the more likely it was to perceive the financial added value of stock management.

### Non-participants in VHHM

The respondents who have not participated in VHHM so far are an interesting target group for offering VHHM service in the future. Thus, one-third of the farmers said that there was a need, and only <10% denied this. More than half of the farmers were interested in support, especially in the areas of fertility and udder health. One-third of the dairy farmers would also be willing to pay the GOT hourly rate for consulting. With this in mind, veterinarians should realize that they can more actively promote VHHM, and farms that are not currently receiving support may just be doing so out of unawareness. On the contrary, the widespread growing shortage of veterinarians could also contribute to the problem, and willing farms cannot be adequately served. All others may not have had any contact with veterinary herd advice and therefore do not know the value of this service. Based on these values, the two groups crystallized in the latent class analysis. In group 2, one out of five dairy farmers provided a receptive target for VHHM. They tend to be larger in number and dissatisfied with their current veterinarian (Table 12) but are also willing to pay for consultative services. The root of this initially contradictive mindset is hard to assess but might be caused by either their own experience with previous veterinarians and through experience exchange with colleagues or the nationwide lack of large animal veterinarians with an offer of VHHM programs. Furthermore, a comparison of these farms with VHHM-participating farms shows, they are more similar in size than those, that are not interested in participating in VHHM (Table 12). Veterinarians should identify these farms and actively invest energy in marketing VHHM (24). As proven before, veterinarians lack of active promotion of their VHHM services (17).

## Conclusion

In summary, the prevalence of VHHM in Germany in this small-scale study was 50%, whereas the overall reason for participating in VHHM was the dairy farmers' interest in the health of their animals. Many of the VHHM participants, mostly running larger herds with higher milk yields, joined this program to achieve herd fertility improvement, while non-participants were divided into those who would consider making use of a service and those who had no interest in participation. The most mentioned critical point was the costs related to the VHHM.

The overall satisfaction of German dairy farmers with their veterinarians was good, while the overall satisfaction rate of VHHM farms was better than that of non-VHHM farms; therefore, this rating provides a suitable basis for further cooperation.

Proactive farm support becomes unavoidable simply because of the recent changes in law. Ideally, the topics covered would extend further than herd fertility and udder health and would increasingly include claw health, young stock rearing, and animal welfare. If the veterinary profession will take advantage of this potential, for example, by cost-benefit analyses and written, farm-specific objectives, the associated benefits could be clearly presented. The VHHM could be expanded according to its intention to provide holistic support to the farm. In addition, this could facilitate future accounting for time invested away from the farm and demonstrate that the VHHM can add value to all sizes of farms. If veterinarians identify the highly receptive portion among non-VHHM farms, this provides a grateful target audience for a VHHM offering.

## Data Availability Statement

The raw data supporting the conclusions of this article will be made available by the authors, without undue reservation.

## Ethics Statement

In this study, no personal or sensible data was collected. Participation was voluntary and anonymous. Before starting the questionnaire, participants perceived detailed information about the aims of the study and how the data were evaluated. Consent was actively given by each participant. We refrained from seeking approval from an Ethics Committee as this was in line with German and European data protection laws.

## Author Contributions

JR conceived and designed the study, developed the theoretical framework, and implemented it in a preliminary model and questionnaire. Statistical preliminary considerations and statistical analyses were performed in close cooperation with RM and KJ. JR drafted and revised the manuscript. RM, K-EM, and CT-R supervised and supported the project at each point of the development, conduction, statistical evaluation, and during the paper-writing process. All authors contributed to the article and approved the submitted version.

## Conflict of Interest

The authors declare that the research was conducted in the absence of any commercial or financial relationships that could be construed as a potential conflict of interest.

## Publisher's Note

All claims expressed in this article are solely those of the authors and do not necessarily represent those of their affiliated organizations, or those of the publisher, the editors and the reviewers. Any product that may be evaluated in this article, or claim that may be made by its manufacturer, is not guaranteed or endorsed by the publisher.

## Abbreviations

AFC: age at first calving
AMS: automatic milking system
BTSCC: bulk tank somatic cell count
DIM: days in milk
EU: European Union
FTE: full-time equivalent
GOT: German Scale of Veterinary Fees
MLP: German DHI testing
QM: German Quality Management Milk
RR: replacement rate
SWOT, strengths, weaknesses, opportunities: and threats
VHHM: Veterinary Herd Health Management
SD: Standard Deviation.
